# Expanding the reach of commercial cell therapies requires changes at medical centers

**DOI:** 10.1186/s12967-024-04966-6

**Published:** 2024-02-19

**Authors:** David F. Stroncek, Nan Zhang, Jiaqiang Ren, Rob Somerville, Anh Dinh

**Affiliations:** The Center for Cellular Engineering, Department of Transfusion Medicine, NIH Clinical Center, 10 Center Drive – MSC -1184, Building 10, Room 3C720, Bethesda, MD 20892-1184 USA

**Keywords:** Cell therapies, Cancer immunotherapies, Apheresis

## Abstract

The clinical application of cell therapies is becoming increasingly important for the treatment of cancer, congenital immune deficiencies, and hemoglobinopathies. These therapies have been primarily manufactured and used at academic medical centers. However, cell therapies are now increasingly being produced in centralized manufacturing facilities and shipped to medical centers for administration. Typically, these cell therapies are produced from a patient’s own cells, which are the critical starting material. For these therapies to achieve their full potential, more medical centers must develop the infrastructure to collect, label, cryopreserve, test, and ship these cells to the centralized laboratories where these cell therapies are manufactured. Medical centers must also develop systems to receive, store, and infuse the finished cell therapy products. Since most cell therapies are cryopreserved for shipment and storage, medical centers using these therapies will require access to liquid nitrogen product storage tanks and develop procedures to thaw cell therapies. These services could be provided by the hospital pharmacy or transfusion service, but the latter is likely most appropriate. Another barrier to implementing these services is the variability among providers of these cell therapies in the processes related to handling cell therapies. The provision of these services by medical centers would be facilitated by establishing a national coordinating center and a network of apheresis centers to collect and cryopreserve the cells needed to begin the manufacturing process and cell therapy laboratories to store and issue the cells. In addition to organizing cell collections, the coordinating center could establish uniform practices for collecting, labeling, shipping, receiving, thawing, and infusing the cell therapy.

Academic medical centers have been involved with cell therapies for many years. Many have developed laboratories to process hematopoietic stem cells (HSCs) for transplantation. Some of these laboratories also manufacture advanced cell therapies, such as cellular cancer immunotherapies, gene therapies for inherited immune deficiencies or hemoglobinopathies, and virus-specific T-cells [1]. These therapies include, but are not limited to, gene-corrected HSCs, chimeric antigen receptor (CAR) T-cells, T-cell receptor-engineered T-cells. Many laboratories are involved with both types of activities. Recently, a third cell therapy activity has emerged for medical centers; handling advanced cell and gene therapies manufactured by companies or other academic centers [2]. These therapies may be allogeneic cells that can be given to multiple eligible recipients, but most are autologous cell therapies manufactured from a patient’s own cells.

Access to cell therapies manufactured offsite is essential for providing comprehensive patient care. At hospitals that currently handle these products, an increasing number of patients are receiving these treatments. For allogeneic cell therapies, hospitals must receive the cell therapy from the laboratory manufacturing the cells, store the cells until they are needed, and issue the cells to the patient. Many of these allogeneic cell therapies are cryopreserved, which requires the hospital to store them in liquid nitrogen freezers until the patient is to be treated, when the cell therapy is thawed and distributed.

For single-center clinical trials using cell therapies manufactured at an academic center, generally, all activities take place onsite. The apheresis procedure to collect the cellular starting material, the manufacturing process, and the storage and infusion of the final product, all take place within the medical center (Fig. 1A). When manufacturing occurs offsite, autologous peripheral blood mononuclear cells or peripheral blood stem cells are collected by apheresis from the patients and, if necessary, are cryopreserved. The cells are sent to a company or academic medical center laboratory that produces the cell therapy (Fig. 1B). Once manufacturing of the cell therapy is complete, the product is generally cryopreserved. If the product meets all quality specifications and lot release criteria, it is shipped to the healthcare center where it is stored until it is administered to the patient.

**Fig. 1:**
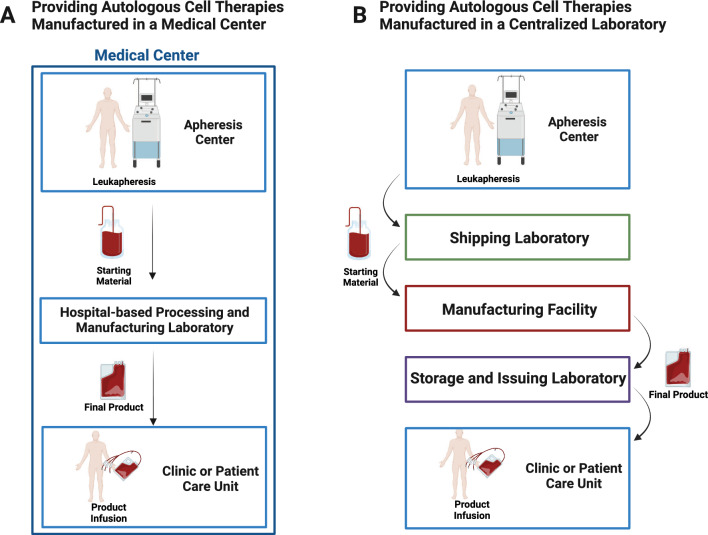
Clinical application of cell therapies. Some medical centers manufacture autologous cell and gene therapies to treat patients at their center, however, many medical centers are using autologous cell therapies manufactured offsite. **A** When cell therapies are manufactured onsite the cells used to begin the manufacturing process are collected in an apheresis center and they are sent to the cell processing laboratory where the cell therapy is manufactured. After manufacturing is complete, the laboratory issues the cell therapy to the patient care unit where it is infused into the patient. **B** When cell therapies are manufactured offsite, the cells used to begin manufacturing are collected in an apheresis unit, the cells are sent to the medical center’s laboratory which ships the cells to the centralized manufacturing laboratory. The medical center laboratory may have to cryopreserve the cells before shipping them to the centralized laboratory. After the centralized laboratory completes the manufacturing of the cells the cell therapy is sent to hospital-based laboratory where it is stored until it is issued to the patient care unit and administered to the patient. (Created with Biorender)

For autologous cell therapies, hospitals must have systems in place to collect, label and ship cells to the laboratory which will manufacture the cells. After the collection, many companies require that laboratory assays be performed on the cellular starting material, including cell counts, flow cytometry analyses, and sterility testing as needed. There are many variations of processing, cryopreservation, and analysis among different clinical therapies. Having streamlined procedures would be beneficial for clinical laboratories in terms of maintaining training and scheduling. In addition, many companies require that the cells undergo minor manipulations and possibly cryopreservation prior to shipping. In hospitals with cell processing facilities, in most cases, the cell processing laboratories are handling these products. For hospitals without cell processing laboratories, acquiring the knowledge and resources to handle these therapies is critical and restricts these therapies to larger institutions. Of note, some hospital-based cell processing facilities may only be able to perform cell collection but do not have the capability or capacity to cryopreserve the cells. Hence, the cells must be shipped to a separate qualified facility for cryopreservation. After that, the cryopreserved cells are shipped to the manufacturing facility. The shipment of cells between these facilities is performed by dedicated cold-chain couriers. Under these circumstances, a well-orchestrated communication mechanism among all relevant parties must be in place to reduce the risk of logistical issues and maintain the quality of the collected cells. Redundancies must also be considered to accommodate unpredictable conditions, such as inclement weather and air flight changes.

While some healthcare centers have apheresis centers to collect the cellular starting material to begin the manufacturing process, many do not. Healthcare centers without apheresis centers could contract with an accredited organization to collect and ship the cells to the manufacturing laboratory. For handling autologous cell therapies manufactured offsite by hospitals with apheresis centers but no cell processing laboratories, the center could collect the cells. A hospital laboratory, such as the transfusion medicine laboratory, could send the cells to the manufacturing laboratory. The transfusion medicine service may need to collaborate with the hospital laboratory medicine department to perform some laboratory analysis of the cells collected by apheresis. The analytical results are important for assessing and controlling critical process and quality parameters for cell therapy manufacturing, such as cell concentration and number, viability, and cell subset percentages. The companies may also have risk assessment procedures in place to omit the sterility testing, or start the manufacturing process without waiting for the final sterility testing results, which may range from 7 to 14 days.

Another pivotal aspect is the management of the finished cell therapy product. When cell therapy manufacturing is complete, the hospital pharmacy or transfusion service should be able to receive, store, and, if necessary, thaw manufactured autologous cell therapy products. However, there are some barriers to completing these tasks. Most of these products are stored cryopreserved in liquid nitrogen either in the vapor phase of qualified liquid nitrogen freezers or in qualified shippers rather than mechanical freezers to prevent product loss due to mechanical freezer failure or electrical power loss. Generally, pharmacies do not have the knowledge, skills, and equipment required for liquid nitrogen storage and thawing of cell therapy products. In contrast, transfusion services routinely handle cryopreserved products stored in liquid nitrogen and routinely thaw and issue blood products. While either the pharmacy or transfusion medicine service could theoretically receive and issue these products, transfusion services laboratories may be better positioned to handle these cell therapies. When external entities manufacture these cells, the final product received may be labeled with different patient- and product-related identifiers compared to the cell therapies developed and manufactured by in-house cell processing laboratories. To maintain vein to vein traceability from collection to infusion, it is essential a rigorous chain of custody and chain of identity procedure is established and to provide proper documentation and labels.

Allogeneic products are generally produced in large lots so that one lot of cells can be used to treat multiple patients. Patients treated with these therapies may often receive multiple treatments. When these allogeneic cells are issued to the patient, the lot number of these issued cells must be documented. These attributes make universal recipient cell therapies similar to pharmaceutical products. In fact, some companies refer to these cell therapies as “drug products.” These products could also be received, stored, and, if necessary, thawed by either the transfusion medicine service or pharmacy. Most of these allogeneic products do not require matching between the recipient’s human leukocyte antigen (HLA) antigens with those of the allogeneic donor, but some require at least partial histocompatibility antigen matching of the allogenic product and the cell therapy recipient. In these cases, the hospital's HLA laboratory or transfusion service will need to be involved.

To advance the field and increase access to these therapies, stakeholders, including healthcare professionals, require additional education in this area. There are several unique aspects concerning handling these products, and healthcare professionals must acquire the skills and knowledge to do so. Being well-informed will allow physicians to collaborate optimally with hospital leadership to develop the infrastructure to support these products. In addition, providers must know how to recognize the complications associated with the infusion of these products, manage these complications, and, if necessary, report these complications to the manufacturer and regulatory agencies. Medical professional societies are well-positioned to become involved with educating laboratory professionals and physicians.

Handling these products is more challenging than anticipated, given the lack of uniform practices for shipping, storing, thawing, and infusing these products. Collaboration among stakeholders, such as manufacturing groups, medical centers, and accrediting bodies, is needed to develop standard practices or, at the very least, best practices. The use of standard practices would reduce training and labor requirements, as well as mitigate the risk for making errors.

Since many hospitals do not have apheresis centers, the development of a coordinating center and a national network of apheresis collection centers would be a significant advancement. This organization could coordinate the collection of the autologous cells used to begin the manufacturing process and the various transportation activities, such as the shipment of the cellular starting material to the cryopreservation facility or the manufacturing laboratory, and, finally, the shipment of the final product to the hospital treating the patient. Furthermore, the coordinating center could develop a set of uniform policies and practices that include shipping, storage, labeling, thawing, infusing, reporting infusion reactions and documenting all of these activities.

In some ways, the current state of this field is reminiscent of HSC transplantation using unrelated donors 35 years ago. At that time, few academic centers were involved with unrelated donor HSC transplantation because the process of finding donors and collecting marrow was difficult. Each center performing these transplants was required to reach out to multiple concurrent small and independent registries of HLA-typed individuals willing to donate marrow for transplantation to a stranger. If an HLA-matched donor was identified for a patient needing a transplant, the transplant center had to work with the specific donor center to arrange the marrow collection. Each registry of HLA-typed donors also had different policies concerning the mechanisms to search for a donor and for collecting marrow. Consequently, the resources, skills, and time required of transplant centers limited the number of unrelated donor transplants they could perform and prevented smaller centers from performing unrelated donor transplants.

This situation was resolved by creating national registries of unrelated donors [3]. This allowed transplant centers to submit one request to search all HLA-typed potential donors in an entire country to find matched donor(s) for a specific patient needing a transplant. The network also arranged for the marrow collection and developed uniform policies and practices. The National Marrow Donor Program (NMDP) has since become the national registry and practice-developing organization for the United States [3]. The development of the NMDP and other national registries worldwide has allowed for the remarkable growth of unrelated donor transplants [4]. Similar to how the NMDP addressed the logistical barriers that hindered access to unrelated donor transplants, an analogous organization focused on cellular therapy product handling logistics, policies, and practices may expand the reach of these therapies and advance the field.

In summary, cell therapies for cancer and other indications are very promising. The number of available products available and in development is increasing. Access to these treatments and the number of patients that could be treated are limited, in part, by logistical challenges of handling these products. Efforts are needed to unify procedures and to develop cell collection and shipping networks, and a coordinating center; this will provide expertise and standard operating procedures, allowing smaller healthcare centers, many of which serve underrepresented communities, to offer these therapies to their patients.

## Data Availability

Not applicable.

## Abbreviations

CAR: Chimeric antigen receptor T-cells
HSCs: Hematopoietic stem cells
HLA: Human leukocyte antigens
NMDP: National marrow donor program

## References

[1] Saez-Ibañez AR, Upadhaya S, Partridge T, Shah M, Correa D, Campbell J (2022). Landscape of cancer cell therapies: trends and real-world data. Nat Rev Drug Discov.

[2] Chen LN, Collins-Johnson N, Sapp N, Pickett A, West K, Stroncek DF, Panch SR (2019). How do I structure logistic processes in preparation for outsourcing of cellular therapy manufacturing?. Transfusion.

[3] McCullough J, Hansen J, Perkins H, Stroncek D, Bartsch G (1989). The National Marrow Donor Program: how it works, accomplishments to date. Oncology.

[4] Stroncek D, Bartsch G, Perkins HA, Randall BL, Hansen JA, McCullough J (1993). The national marrow donor program. Transfusion.

